# A design criterion for symmetric model discrimination based on flexible nominal sets

**DOI:** 10.1002/bimj.201900074

**Published:** 2020-01-20

**Authors:** Radoslav Harman, Werner G. Müller

**Affiliations:** ^1^ Faculty of Mathematics, Physics and Informatics Comenius University Bratislava Slovakia; ^2^ Department of Applied Statistics Johannes Kepler University Linz Linz Austria

**Keywords:** discrimination experiments, exact designs, flexible nominal sets, nonlinear regression

## Abstract

Experimental design applications for discriminating between models have been hampered by the assumption to know beforehand which model is the true one, which is counter to the very aim of the experiment. Previous approaches to alleviate this requirement were either symmetrizations of asymmetric techniques, or Bayesian, minimax, and sequential approaches. Here we present a genuinely symmetric criterion based on a linearized distance between mean‐value surfaces and the newly introduced tool of flexible nominal sets. We demonstrate the computational efficiency of the approach using the proposed criterion and provide a Monte‐Carlo evaluation of its discrimination performance on the basis of the likelihood ratio. An application for a pair of competing models in enzyme kinetics is given.

## 1 INTRODUCTION

Besides optimization and parameter estimation, discrimination between rival models has always been an important objective of an experiment, and, therefore, of the optimization of experimental design. The crucial problem is that one typically cannot construct an optimal model‐discrimination design without already knowing which model is the true one, and what are the true values of its parameters. In this respect, the situation is analogous to the problem of optimal experimental design for parameter estimation in nonlinear statistical models (e.g., Pronzato & Pazman, 2014), and many standard techniques can be used to tackle the dependence on the unknown characteristics: local, Bayesian, minimax, and sequential approaches, as well as their various combinations.

A big leap from initial ad hoc methods for model discrimination (see Hill, 1978, for a review) was Atkinson and Fedorov (1975) who introduced *T*‐optimality, derived from the likelihood‐ratio test under the assumption that one model is true and its parameters are fixed at nominal values chosen by the experimenter. There, maximization of the noncentrality parameter is equivalent to maximizing the power of the likelihood‐ratio test for the least favorable parameter of the model assumed to be wrong. Thus, *T*‐optimality can be considered a combination of a localization and a minimax approach.

When the models are nested and (partly) linear, *T*‐optimality can be shown to be equivalent to $D_{s}$‐optimality for a single parameter that embodies the deviations from the smaller model (see, e.g., Dette & Titoff, 2009; Stigler, 1971). For this setting the optimal design questions are essentially solved and everything hinges on the asymmetric nature of the NP‐lemma with respect to the null‐ and alternative hypotheses. However, for a nonnested case the design problem itself is often inherently symmetric with respect to the exchangeability of the compared models and it is the purpose of the experiment to decide which of those two different models is true.

The aim of this paper is to solve the discrimination design problem in a symmetric way focusing on nonnested models. Thus, standard methods that are inherently asymmetric like *T*‐optimality, albeit being feasible, are not a natural choice. We further suppose that we do not use the full prior distribution of the unknown parameters of the models, which rules out Bayesian approaches such as Felsenstein (1992) and Tommasi and López‐Fidalgo (2010). Nevertheless, as we will make more precise in the next section, we will utilize what can be perceived as a specific kind of prior knowledge about the unknown parameters, extending the approach of local optimality. Our goal is to provide a lean, computationally efficient and scalable method as opposed to the heavy machinery recently employed in the computational statistics literature, for example, Hainy, Price, Restif, and Drovandi (2018). Furthermore, we strive for practical simplicity, which at first prohibits sequential (see Buzzi‐Ferraris & Forzatti, 1983; Müller & Ponce De Leon, 1996; Schwaab et al., 2006) or sequentially generated (see Vajjah & Duffull, 2012) designs.

A standard solution to the symmetric discrimination design problem is to employ symmetrizations of asymmetric criteria such as compound *T*‐optimality, which usually depend on some weighting chosen by the experimenter. Also the minimax strategy recently presented in Tommasi, Martín‐Martín, and López‐Fidalgo (2016) is essentially a symmetrization. Moreover, the usual minimax approaches lead to designs that completely depend on the possibly unrealistic extreme values of the parameter space and their calculation again demands enormous computational effort.

As the closest in spirit to our approach could be considered a proposal for linear models in section 4.4 of Atkinson and Fedorov (1975) and its extension in Fedorov and Khabarov (1986) which, however, was not taken up by the literature. The probable reason is that it involves some rather arbitrary restrictions on the parameters as well as taking an artificial lower bound to convert it into a computationally feasible optimization problem.

For expositional purposes we will now restrict ourselves to a rather specific design task but will discuss possible extensions at the end of the paper.

Let X≠∅ be a finite design space and let D be a design on X, that is, a vector of design points $x_{1},\ldots ,x_{n}\in X$, where *n* is the chosen size of the experiment. Hence, in the terminology of the theory of optimal experimental design, we will work with *exact* designs. We will consider discrimination between a pair of nonlinear regression models

$$\begin{array}{ccc} y_{i} & = & \eta_{0}\left(\theta_{0},x_{i}\right)+\varepsilon_{i},\;\;i=1,\ldots ,n,\;\text{and}\\ y_{i} & = & \eta_{1}\left(\theta_{1},x_{i}\right)+\varepsilon_{i},\;\;i=1,\ldots ,n, \end{array}$$

where $y_{1},\ldots ,y_{n}$ are observations, $\eta_{0}:\Theta_{0}\times X\to R$, $\eta_{1}:\Theta_{1}\times X\to R$ are the mean‐value functions, $\Theta_{0}\subseteq R^{m_{0}}$, $\Theta_{1}\subseteq R^{m_{1}}$ are parameter spaces with nonempty interiors int(Θ_0_), int(Θ_1_), and $\varepsilon_{1},\ldots ,\varepsilon_{n}$ are unobservable random errors. For both k=0,1 and any x∈X, we will assume that the functions $\eta_{k}\left(\cdot ,x\right)$ are differentiable on $\mathrm{int}(\Theta_{k})$; the gradient of $\eta_{k}\left(\cdot ,x\right)$ in $\theta_{k}\in \mathrm{int}\left(\Theta_{k}\right)$ will be denoted by $\nabla \eta_{k}\left(\theta_{k},x\right)$. Our principal assumption is that one of the models is true but we do not know which, that is, for k=0 or for k=1 there exists ${\bar{\theta }}_{k}\in \Theta_{k}$ such that $y_{i}=\eta_{k}\left({\bar{\theta }}_{k},x_{i}\right)+\varepsilon_{i}$.

Let the random errors be i.i.d. $N(0,\sigma^{2})$, where $\sigma^{2}\in \left(0,\infty \right)$. The assumption of the same variances of the errors for both models is plausible if, for instance, the errors are due to the measurement device and hence do not significantly depend on the value being measured. The situation with different error variances requires a more elaborate approach, compared with Fedorov and Pázman (1968).

Eventually we are aiming not just at achieving some high design efficiencies with respect to our newly proposed criterion, but also want to test its usefulness in concrete discrimination experiments, that is, the probability that using our design we arrive at the correct decision about which model is the true one. So, to justify our approach numerically, we require a model‐discrimination rule that will be used after all observations based on the design D are collected for evaluational purposes.

The choice of the best discrimination rule based on the observations is generally a nontrivial problem. However, it is natural to compute the maximum likelihood estimates ${\hat{\theta }}_{0}$ and ${\hat{\theta }}_{1}$ of the parameters under the assumption of the first and the second model, respectively, and then base the decision on whether

(1) $$\frac{L({\hat{\theta }}_{0}|{\left(y_{i}\right)}_{i=1}^{n})}{L({\hat{\theta }}_{1}|{\left(y_{i}\right)}_{i=1}^{n})}<>1,$$

that is, the likelihood ratio being smaller or greater than 1, or perhaps more simply whether $\log L\left({\hat{\theta }}_{0}\right)-\log L\left({\hat{\theta }}_{1}\right)<>0$. Under the normality, homoskedasticity, and independence assumptions, this decision is equivalent to a decision based on the proximity of the vector ${\left(y_{i}\right)}_{i=1}^{n}$ of observations to the vectors of estimated mean values ${\left(\eta_{0}\left({\hat{\theta }}_{0},x_{i}\right)\right)}_{i=1}^{n}$ and ${\left(\eta_{1}\left({\hat{\theta }}_{1},x_{i}\right)\right)}_{i=1}^{n}$.

For the case $m_{0}\neq m_{1}$ to counterbalance favoring models with greater number of parameters, Cox (2013) recommends instead the use of $L\left({\hat{\theta }}_{0}\right)/L\left({\hat{\theta }}_{1}\right){\left(e^{m_{1}}/e^{m_{0}}\right)}^{n/\tilde{n}}$, which corresponds to the Bayesian information criterion; see Schwarz (1978). Here $\tilde{n}$ corresponds to the number of observations in a real or fictitious prior experiment. For the sake of simplicity however, we will now restrict ourselves to the case of $m:=m_{0}=m_{1}$. Note that for the evaluational purposes we are taking a purely model selection based standpoint. More sophisticated testing procedures for instance allowing both models to be rejected based on the pioneering work of Cox (1961) are reviewed and outlined in Pesaran and Weeks (2007).

Let $x_{1},\ldots ,x_{n}\in X$ and let $D=(x_{1},\ldots ,x_{n})$ be the design used for the collection of data prior to the decision, and assume that model η_0_ is true, with the corresponding parameter value ${\bar{\theta }}_{0}$. Note that this comes without loss of generality and symmetry as we can equivalently assume model η_1_ to be true. Then, the probability of the correct decision based on the likelihood ratio is equal to

(2) $$P\left[\min_{\theta_{0}\in \Theta_{0}}\sum_{i=1}^{n}{\left(\eta_{0}\left(\theta_{0},x_{i}\right)-y_{i}\right)}^{2}\leq \min_{\theta_{1}\in \Theta_{1}}\sum_{i=1}^{n}{\left(\eta_{1}\left(\theta_{1},x_{i}\right)-y_{i}\right)}^{2}\right],$$

where ${\left(y_{i}\right)}_{i=1}^{n}$ follows the normal distribution with mean ${\left(\eta_{0}\left({\bar{\theta }}_{0},x_{i}\right)\right)}_{i=1}^{n}$ and covariance $\sigma^{2}I_{n}$.

Clearly, probability (2) depends on the true model, the unknown true parameter, and also on the unknown variance of errors. Even if these parameters were known, the probability of the correct classification would be very difficult to compute for a given design because this requires a combination of high‐dimensional integration and multivariate nonconvex optimization. Therefore, it is practically impossible to directly optimize the design based on formula (2). However, we can simplify the problem by constructing a lower bound on (2) which does not depend on unknown parameters and is relatively much simpler to maximize with respect to the choice of the design. The bound based on the distance $d(E_{0},E_{1})$, where $E_{j}$ is the set of all possible mean values of the observations under the model *j*, j=0,1, and *d*(., .) denotes the infimum distance between all pairs of elements of two sets, is developed as follows.

Consider a fixed experimental design $\left(x_{1},\ldots ,x_{n}\right)$, and denote $y:={\left(y_{i}\right)}_{i=1}^{n}$, $\eta_{j}\left(\theta_{j}\right):={\left(\eta_{j}\left(\theta_{j},x_{i}\right)\right)}_{i=1}^{n}$ for j=0,1. Note that we can express (2) as $P[d\left(E_{0},y\right)\leq d\left(E_{1},y\right)]$. Now, let R=∥ε∥, where $\varepsilon =y-\eta_{0}\left({\bar{\theta }}_{0}\right)$, be the norm of the vector of errors. Assuming $R\leq d(E_{0},E_{1})/2$ we obtain

$$\begin{array}{ccc} & & d\left(E_{0},E_{1}\right)\leq d\left(\eta_{0}\left({\hat{\theta }}_{0}\right),\eta_{1}\left({\hat{\theta }}_{1}\right)\right)\leq d\left(y,\eta_{0}\left({\hat{\theta }}_{0}\right)\right)+d\left(y,\eta_{1}\left({\hat{\theta }}_{1}\right)\right)\\ & & \;\leq d\left(y,\eta_{0}\left({\bar{\theta }}_{0}\right)\right)+d\left(y,\eta_{1}\left({\hat{\theta }}_{1}\right)\right)=R+d\left(y,\eta_{1}\left({\hat{\theta }}_{1}\right)\right)\leq d\left(E_{0},E_{1}\right)/2+d\left(y,\eta_{1}\left({\hat{\theta }}_{1}\right)\right), \end{array}$$

which implies $d\left(E_{0},E_{1}\right)/2\leq d\left(y,\eta_{1}\left({\hat{\theta }}_{1}\right)\right)$ and consequently

$$d\left(E_{0},y\right)=d\left(y,\eta_{0}\left({\hat{\theta }}_{0}\right)\right)\leq d\left(y,\eta_{0}\left({\bar{\theta }}_{0}\right)\right)=R\leq d\left(E_{0},E_{1}\right)/2\leq d\left(y,\eta_{1}\left({\hat{\theta }}_{1}\right)\right)=d\left(E_{1},y\right).$$

Thus, the event $\left[R\leq d\left(E_{0},E_{1}\right)/2\right]$ implies the event $\left[d\left(E_{0},y\right)\leq d\left(E_{1},y\right)\right]$, that is, (2) can be bounded from below by

(3) $$P\left[R\leq d(E_{0},E_{1})/2\right].$$

To make (2) as high as possible, it makes sense to maximize (3), that is, maximize $d(E_{0},E_{1})$, which depends on the underlying experimental design. Although this maximization is much simpler than maximizing (2) directly, it still generally requires nonconvex multidimensional optimization at each iteration of the maximization procedure, which is impractical for computing exact optimal designs. A realistic approach must be numerically feasible and address the problem of the dependence of the design on unknown true model parameters, which we will achieve by rapidly computable approximation of $d(E_{0},E_{1})$ through linearization, as will be explained in the following section.

### 1.1 Example 1: A motivating example

**Figure 1:**
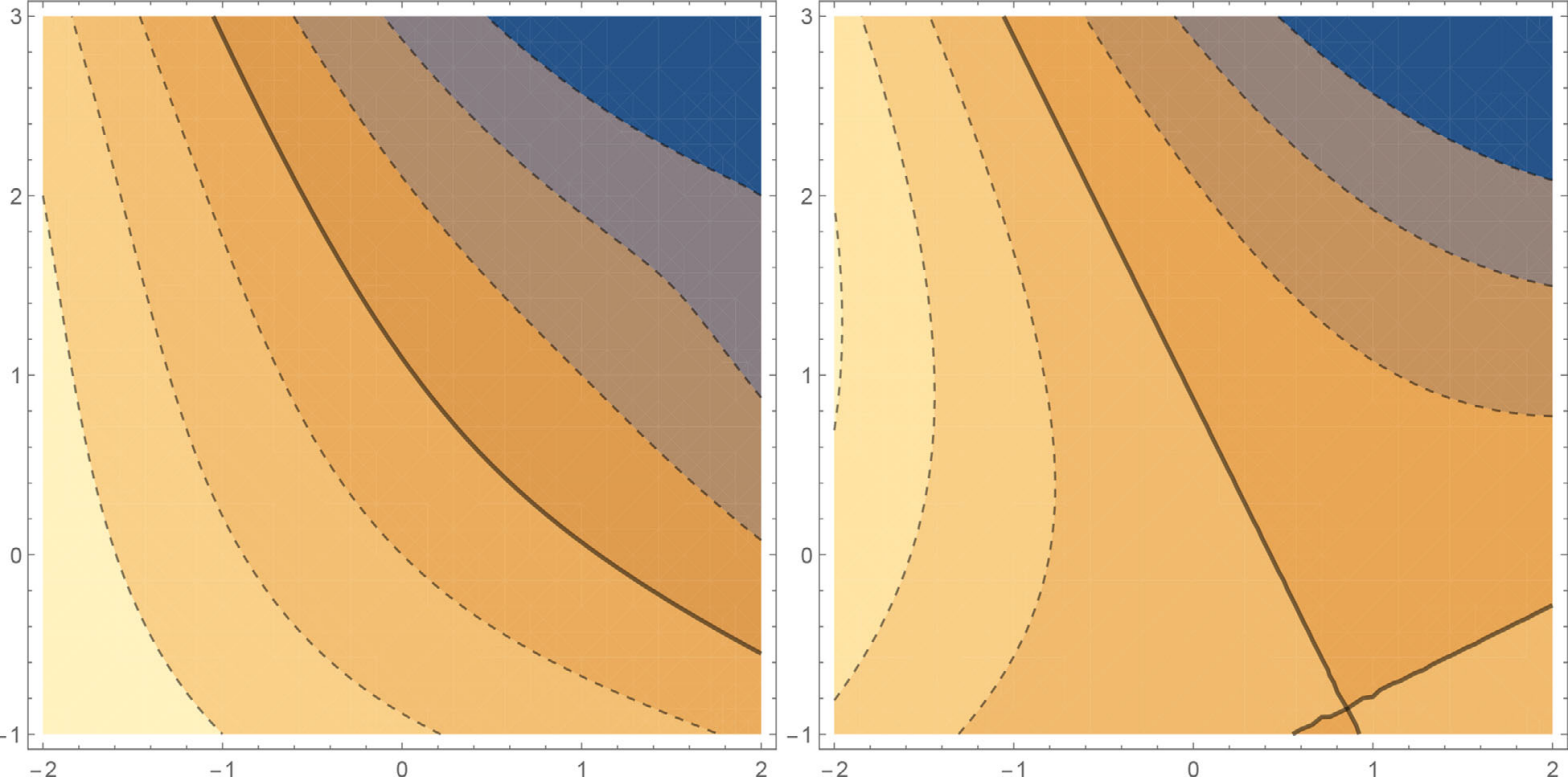
Left panel: contour plot of $\log L\left({\hat{\theta }}_{0}\right)-\log L\left({\hat{\theta }}_{1}\right)$ for Example 1, solid line corresponds to 0, horizontal *y*_1_, vertical *y*_2_; right panel: corresponding contour plot for the model η_1_ linearized at ${\tilde{\theta }}_{1}=1$

Let $\eta_{0}\left(\theta_{0},x\right)=\theta_{0}x$ and $\eta_{1}\left(\theta_{1},x\right)=e^{\theta_{1}x}$. Furthermore, for the moment we assume just two observations $y_{1},y_{2}$ at fixed design points $x_{1}=-1$ and $x_{2}=1$, respectively. In this case evidently ${\hat{\theta }}_{0}=\frac{y_{2}-y_{1}}{2}$ and ${\hat{\theta }}_{1}$ is the solution of $2e^{-\theta }\left(y_{1}-e^{-\theta }\right)-2e^{\theta }\left(y_{2}-e^{\theta }\right)=0$, which for $-2\leq y_{1}\leq 2$ is the log root of the polynomial $\gamma^{4}-\gamma^{3}y_{2}+\gamma y_{1}-1$. Figure 1 displays the log‐likelihood‐ratio contours for the original and linearized models and it is obvious that the former are nonconvex and complex while the latter are much simpler, convex, and do approximate fairly well for a wide range of responses. Note that while this example is for a fixed design it motivates why the linearizations can serve as the cornerstones of our design method as will become clearer in the following sections.

## 2 THE LINEARIZED DISTANCE CRITERION

We suggest an extension of the idea of local optimality used for nonlinear experimental design. Let ${\tilde{\theta }}_{0}\in \mathrm{int}\left(\Theta_{0}\right)$ and ${\tilde{\theta }}_{1}\in \mathrm{int}\left(\Theta_{1}\right)$ be nominal parameter values, which satisfy the basic *discriminability condition* $\eta_{0}\left({\tilde{\theta }}_{0},x\right)\neq \eta_{1}\left({\tilde{\theta }}_{1},x\right)$ for some x∈X. Let us introduce regions ${\tilde{\Theta }}_{0}\subseteq \mathrm{int}\left(\Theta_{0}\right)\subseteq R^{m}$ and ${\tilde{\Theta }}_{1}\subseteq \mathrm{int}\left(\Theta_{1}\right)\subseteq R^{m}$ containing ${\tilde{\theta }}_{0}$ and ${\tilde{\theta }}_{1}$; we will consequently call ${\tilde{\Theta }}_{0}$ and ${\tilde{\Theta }}_{1}$ *flexible nominal sets*. It is evident that optimal designs depend on the parameter spaces in the same way as on our flexible nominal sets (cf. Dette, Melas, & Shpilev, 2013), but the latter will not be considered fixed like the parameter spaces Θ_0_ and Θ_1_. A novelty of our procedure is that we use these sets as a tuning device.

Let $D=(x_{1},\ldots ,x_{n})$ be a design. Let us perform the following particular linearization of Model $\eta_{k=0,1}$ in ${\tilde{\theta }}_{k}$:

$${\left(y_{i}\right)}_{i=1}^{n}\approx F_{k}\left(D\right)\theta_{k}+a_{k}\left(D\right)+\varepsilon ,$$

where $F_{k}\left(D\right)$ is the n×m matrix given by

$$F_{k}\left(D\right)={\left(\nabla \eta_{k}\left({\tilde{\theta }}_{k},x_{1}\right),\ldots ,\nabla \eta_{k}\left({\tilde{\theta }}_{k},x_{n}\right)\right)}^{T},$$

$a_{k}\left(D\right)$ is the *n*‐dimensional vector

$$a_{k}\left(D\right)={\left(\eta_{k}\left({\tilde{\theta }}_{k},x_{i}\right)\right)}_{i=1}^{n}-F_{k}\left(D\right){\tilde{\theta }}_{k},$$

and $\varepsilon ={\left(\varepsilon_{1},\ldots ,\varepsilon_{n}\right)}^{T}$ is a vector of independent $N(0,\sigma^{2})$ errors.

Note that for the proposed method the vector $a_{k}\left(D\right)$ plays an important role and, although it is known, we cannot subtract it from the vector of observations, as is usual when we linearize a single nonlinear regression model. However, if $\eta_{k}$ corresponds to the standard linear model then $a_{k}\left(D\right)=0_{n}$ for any D.

### 2.1 Definition of the δ criterion

**Figure 2:**
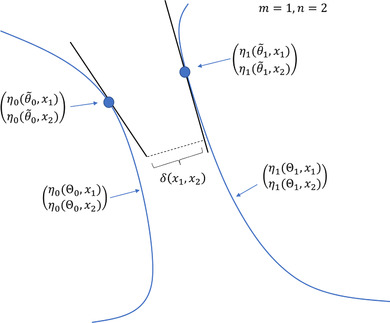
Illustrative graph for the definition of δ(D) for two one‐parameter models ($\Theta_{0},\Theta_{1}\subseteq R$) and a design of size two ($D=(x_{1},x_{2})$). The line segments correspond to the sets $\left\{a_{0}\left(D\right)+F_{0}\left(D\right)\theta_{0}:\theta_{0}\in {\tilde{\Theta }}_{0}\right\}$ and $\left\{a_{1}\left(D\right)+F_{1}\left(D\right)\theta_{1}:\theta_{1}\in {\tilde{\Theta }}_{1}\right\}$ for some flexible nominal sets ${\tilde{\Theta }}_{0}$ and ${\tilde{\Theta }}_{1}$

Consider the design criterion

(4) $$\delta \left(D\right)=\underset{\theta_{0}\in {\tilde{\Theta }}_{0},\theta_{1}\in {\tilde{\Theta }}_{1}}{\inf}\delta \left(D|\theta_{0},\theta_{1}\right),\;\text{where}\;$$

(5) $$\delta \left(D|\theta_{0},\theta_{1}\right)=\left\|a_{0}\left(D\right)+F_{0}\left(D\right)\theta_{0}-\left\{a_{1}\left(D\right)+F_{1}\left(D\right)\theta_{1}\right\}\right\|,$$

for $\theta_{0}\in {\tilde{\Theta }}_{0},\theta_{1}\in {\tilde{\Theta }}_{1}$. The criterion δ can be viewed as an approximation of the nearest distance *d* of the mean‐value surfaces of the models, in the neighborhoods of the vectors ${\left(\eta_{0}\left({\tilde{\theta }}_{0},x_{i}\right)\right)}_{i=1}^{n}$ and ${\left(\eta_{1}\left({\tilde{\theta }}_{1},x_{i}\right)\right)}_{i=1}^{n}$; see the illustrative Figure 2.

We will now express the δ‐criterion as a function of the design $D=(x_{1},\ldots ,x_{n})$ represented by the counting measure ξ on X defined as

$$\xi \left(\left\{x\right\}\right):=\#\{i\in \left\{1,\ldots ,n\right\}:x_{i}=x\},\;x\in X,$$

where # means the size of a set. Let $\tilde{\theta }={\left({\tilde{\theta }}_{0}^{T},{\tilde{\theta }}_{1}^{T}\right)}^{T}$. For all x∈X let

$$\begin{array}{ccc} \Delta \eta (\tilde{\theta },x) & := & \eta_{0}\left({\tilde{\theta }}_{0},x\right)-\eta_{1}\left({\tilde{\theta }}_{1},x\right),\\ \nabla \eta (\tilde{\theta },x) & := & {\left(\nabla \eta_{0}^{T}\left({\tilde{\theta }}_{0},x\right),\;-\nabla \eta_{1}^{T}\left({\tilde{\theta }}_{1},x\right)\right)}^{T}. \end{array}$$

For any $\theta_{0}\in {\tilde{\Theta }}_{0}$, $\theta_{1}\in {\tilde{\Theta }}_{1}$ and $\theta ={\left(\theta_{0}^{T},\theta_{1}^{T}\right)}^{T}$ we have

(6) $$\begin{array}{ccc} \delta^{2}\left(D|\theta_{0},\theta_{1}\right) & = & {\left\|a_{0}\left(D\right)+F_{0}\left(D\right)\theta_{0}-\left\{a_{1}\left(D\right)+F_{1}\left(D\right)\theta_{1}\right\}\right\|}^{2}\\ & = & \sum_{i=1}^{n}{\left(\nabla \eta^{T}\left(\tilde{\theta },x_{i}\right)\left(\theta -\tilde{\theta }\right)+\Delta \eta \left(\tilde{\theta },x_{i}\right)\right)}^{2}\\ & = & \int_{X}{\left(\nabla \eta^{T}\left(\tilde{\theta },x\right)\left(\theta -\tilde{\theta }\right)+\Delta \eta \left(\tilde{\theta },x\right)\right)}^{2}d\xi \left(x\right). \end{array}$$

Therefore

(7) $$\delta^{2}\left(D|\theta_{0},\theta_{1}\right)={\left(\theta -\tilde{\theta }\right)}^{T}M\left(\xi ,\tilde{\theta }\right)\left(\theta -\tilde{\theta }\right)+2b^{T}\left(\xi ,\tilde{\theta }\right)\left(\theta -\tilde{\theta }\right)+c\left(\xi ,\tilde{\theta }\right),$$

where

(8) $$M\left(\xi ,\tilde{\theta }\right)=\int_{X}\nabla \eta \left(\tilde{\theta },x\right)\nabla \eta^{T}\left(\tilde{\theta },x\right)d\xi \left(x\right),$$

(9) $$b\left(\xi ,\tilde{\theta }\right)=\int_{X}\Delta \eta \left(\tilde{\theta },x\right)\nabla \eta \left(\tilde{\theta },x\right)d\xi \left(x\right),$$

(10) $$c\left(\xi ,\tilde{\theta }\right)=\int_{X}{\left[\Delta \eta \left(\tilde{\theta },x\right)\right]}^{2}d\xi \left(x\right).$$

The matrix $M(\xi ,\tilde{\theta })$ in Equations (7) and (8) can be recognized as the information matrix for the parameter θ in the linear regression model

(11) $$\begin{array}{ccc} z_{i} & = & \nabla \eta^{T}\left(\tilde{\theta },x_{i}\right)\theta +\varepsilon_{i}\\ & = & {\left[F_{0}\left(D\right),-F_{1}\left(D\right)\right]}_{i\cdot }\theta +\varepsilon_{i};\;i=1,\ldots ,n, \end{array}$$

where ${\left[F_{0}\left(D\right),-F_{1}\left(D\right)\right]}_{i\cdot }$ is the *i*th row of the matrix $\left[F_{0}\left(D\right),-F_{1}\left(D\right)\right]$, with parameter θ and independent, homoskedastic errors $\varepsilon_{1},\ldots ,\varepsilon_{n}$ with mean 0; we will call (11) a *response difference model*.

### 2.2 Computation of the δ criterion value for a fixed design

For a fixed design D, expression (5) shows that $\delta^{2}\left(D|\theta \right)$ is a quadratic function of $\theta ={\left(\theta_{0}^{T},\theta_{1}^{T}\right)}^{T}$. Moreover, both δ(D|θ) and $\delta^{2}\left(D|\theta \right)$ are convex because they are compositions of an affine function of θ and convex functions ∥.∥ and ${∥.∥}^{2}$, respectively. Clearly, if the flexible nominal sets are compact, convex, and polyhedral, optimization (4) can be efficiently performed by specialized solvers for linearly constrained quadratic programming.

Alternatively, we can view the computation of δ(D|θ) as follows. As

$$\delta^{2}\left(D|\theta_{0},\theta_{1}\right)={\left\|\left\{a_{0}\left(D\right)-a_{1}\left(D\right)\right\}-\left[-F_{0}\left(D\right),F_{1}\left(D\right)\right]\theta \right\|}^{2},$$

the minimization in (4) is equivalent to computing the minimum sum of squares for a least squares estimate of θ restricted to $\tilde{\Theta }:={\tilde{\Theta }}_{0}\times {\tilde{\Theta }}_{1}$ in the response difference model with artificial observations

$${\tilde{z}}_{i}={\left\{a_{1}\left(D\right)-a_{0}\left(D\right)\right\}}_{i},\;i=1,\ldots ,n.$$

Thus, if ${\tilde{\Theta }}_{0}={\tilde{\Theta }}_{1}=R^{m}$, the infimum in (4) is attained, and it can be computed using the standard formulas of linear regression in the response difference model. If the flexible nominal sets are compact cuboids, (4) can be evaluated by the very rapid and stable method for bounded variable least squares implemented in the R package bvls; see Stark and Parker (1995) and Mullen (2013).

The following simple proposition collects the analytic properties of a natural analogue of δ defined on the linear vector space Ξ of all finite signed measures on X.

Proposition 2.1 For $\theta_{0}\in {\tilde{\Theta }}_{0}$, $\theta_{1}\in {\tilde{\Theta }}_{1}$ and a finite signed measure ξ on X let $\delta_{app}^{2}\left(\xi |\theta_{0},\theta_{1}\right)$ be defined via formula (6). Then, $\delta_{app}^{2}\left(\cdot |\theta_{0},\theta_{1}\right)$ is linear on Ξ. Moreover, let

$$\delta_{app}^{2}\left(\xi \right):=\underset{\theta_{0}\in {\tilde{\Theta }}_{0},\theta_{1}\in {\tilde{\Theta }}_{1}}{\inf}\delta_{app}^{2}\left(\xi |\theta_{0},\theta_{1}\right).$$

Then, $\delta_{app}^{2}$ is positive homogeneous and concave on Ξ.

Positive homogeneity of $\delta_{app}^{2}$ implies that an *s*‐fold replication of an exact design leads to an *s*‐fold increase of its δ^2^ value. Consequently, a natural and statistically interpretable definition of relative δ‐efficiency of two designs $D_{1}$ and $D_{2}$ is given by $\delta^{2}\left(D_{1}\right)/\delta^{2}\left(D_{2}\right)$, provided that $\delta^{2}\left(D_{2}\right)>0$.

Let D be the set of all *n*‐point designs. A design $D^{*}\in D$ will be called δ‐optimal, if

$$D^{*}\in {\mathrm{argmax}}_{D\in D}\delta \left(D\right).$$

Note that the basic discriminability condition implies that if ${\tilde{\Theta }}_{0}=\left\{{\tilde{\theta }}_{0}\right\}$ and ${\tilde{\Theta }}_{1}=\left\{{\tilde{\theta }}_{1}\right\}$, then $\delta (D^{*})$ is strictly positive. However, for larger flexible nominal sets it can happen that $\delta (D^{*})=0$.

As the evaluation of the δ‐criterion is generally very rapid, the calculation of a δ‐optimal, or nearly δ‐optimal design is similar to that for standard design criteria. For instance, in small problems we can use complete‐enumeration and in larger problems we can employ an exchange heuristic, such as the KL exchange algorithm (see, e.g., Atkinson, Donev, & Tobias, 2007).

Note that the δ‐optimal designs depend not only on η_0_, η_1_, X, *n*, ${\tilde{\theta }}_{0}$, and ${\tilde{\theta }}_{1}$, but also on ${\tilde{\Theta }}_{0}$ and ${\tilde{\Theta }}_{1}$.

### 2.3 Parametrization of flexible nominal sets

For simplicity, we will focus on cuboid flexible nominal sets centered at the nominal parameter values. This choice can be justified by the results of Sidak (1967), in particular if we already have confidence intervals for individual parameters; see further discussion in Section 4. Specifically, we will employ the homogeneous dilations

(12) $${\tilde{\Theta }}_{k}^{\left(r\right)}:=r\left({\tilde{\Theta }}_{k}^{\left(1\right)}-{\tilde{\theta }}_{k}\right)+{\tilde{\theta }}_{k},\;r\in \left[0,\infty \right),\;k=0,1,$$

${\tilde{\Theta }}_{0}^{\left(\infty \right)}:=R^{m}$, ${\tilde{\Theta }}_{1}^{\left(\infty \right)}:=R^{m}$, such that *r* can be considered a tuning (set) parameter governing the size of the flexible nominal sets. In (12), ${\tilde{\Theta }}_{0}^{\left(1\right)}$ and ${\tilde{\Theta }}_{1}^{\left(1\right)}$ are “unit” nondegenerate compact cuboids centered on respective nominal parameters. For any design D and r∈[0,∞], we define

(13) $$\delta_{r}\left(D\right):=\underset{\theta_{0}\in {\tilde{\Theta }}_{0}^{\left(r\right)},\theta_{1}\in {\tilde{\Theta }}_{1}^{\left(r\right)}}{\inf}\delta \left(D|\theta_{0},\theta_{1}\right).$$

Note that for our choice of flexible nominal sets the infimum in (13) is attained. The $\delta_{r}$‐optimal values of the problem will be denoted by

$$o\left(r\right):=\max_{D\in D}\delta_{r}\left(D\right).$$

Proposition 2.2 (a) Let D be a design. Functions $\delta_{r}^{2}\left(D\right)$, $\delta_{r}\left(D\right)$, $o^{2}\left(r\right)$, o(r) are nonincreasing and convex in *r* on the entire interval [0, ∞]. (b) There exists $r^{*}<\infty$, such that for all $r\geq r^{*}$: (i) o(r)=o(∞); (ii) Any $\delta_{\infty }$‐optimal design is also a $\delta_{r}$‐optimal design.

(a) Let D be an *n*‐point design and let $0\leq r_{1}\leq r_{2}\in \left[0,\infty \right]$.

Inequality $\delta_{r_{1}}^{2}\left(D\right)\geq \delta_{r_{2}}^{2}\left(D\right)$ follows from definitions (12) and (13), and inequality $o^{2}\left(r_{1}\right)\geq o^{2}\left(r_{2}\right)$ follows from the fact that a maximum of nonincreasing functions is a nonincreasing function. Monotonicity of $\delta_{r}\left(D\right)$ and o(r) in *r* can be shown analogously.

To prove the convexity of $\delta_{r}^{2}\left(D\right)$ in *r*, let α∈(0,1) and let $r_{\alpha }=\alpha r_{1}+\left(1-\alpha \right)r_{2}$. For all r∈[0,∞], let ${\hat{\theta }}_{r}$ denote a minimizer of $\delta_{r}^{2}\left(D|\cdot \right)$ on ${\tilde{\Theta }}^{\left(r\right)}:={\tilde{\Theta }}_{0}^{\left(r\right)}\times {\tilde{\Theta }}_{1}^{\left(r\right)}$. Convexity of $\delta^{2}\left(D|\theta \right)$ in θ and a simple fact $\alpha {\hat{\theta }}_{r_{1}}+\left(1-\alpha \right){\hat{\theta }}_{r_{2}}\in {\tilde{\Theta }}^{\left(r_{\alpha }\right)}$ yield

$$\begin{array}{c} \alpha \delta_{r_{1}}^{2}\left(D\right)+\left(1-\alpha \right)\delta_{r_{2}}^{2}\left(D\right)=\alpha \delta^{2}\left(D|{\hat{\theta }}_{r_{1}}\right)+\left(1-\alpha \right)\delta^{2}\left(D|{\hat{\theta }}_{r_{2}}\right)\\ \geq \delta^{2}\left(D|\alpha {\hat{\theta }}_{r_{1}}+\left(1-\alpha \right){\hat{\theta }}_{r_{2}}\right)\geq \delta^{2}\left(D|{\hat{\theta }}_{r_{\alpha }}\right)=\delta_{r_{\alpha }}^{2}\left(D\right), \end{array}$$

which proves that $\delta_{r}^{2}\left(D\right)$ is convex in *r*. The convexity of $\delta_{r}\left(D\right)$ in *r* can be shown analogously. The functions *o* ^2^ and *o*, as pointwise maxima of a system of convex functions, are also convex.

(b) For any design D of size *n*, the function $\delta_{\infty }^{2}\left(D|\cdot \right)$ is nonnegative and quadratic on $R^{2m}$, therefore its minimum is attained in some $\theta_{D}\in R^{2m}$. There is only a finite number of exact designs of size *n*, and ${\tilde{\Theta }}^{\left(r\right)}{↑}_{r}R^{2m}$, which means that there exists $r^{*}<\infty$ such that $\theta_{D}\in {\tilde{\Theta }}^{\left(r^{*}\right)}$ for all designs D of size *n*. Let $r\geq r^{*}$. We have

$$o\left(\infty \right)=\max_{D\in D}\min_{\theta \in R^{2m}}\delta_{\infty }\left(D|\theta \right)=\max_{D\in D}\min_{\theta \in {\tilde{\Theta }}^{\left(r\right)}}\delta \left(D|\theta \right)=\max_{D\in D}\delta_{r}\left(D\right)=o\left(r\right),$$

proving (i). Let $D^{\left(\infty \right)}$ be any $\delta_{\infty }$‐optimal *n*‐trial design. The equality (i) and the fact that $\delta_{r}\left(D^{\left(\infty \right)}\right)$ and o(r) are nonincreasing with respect to *r* gives

$$\delta_{r}\left(D^{\left(\infty \right)}\right)\geq \delta_{\infty }\left(D^{\left(\infty \right)}\right)=o\left(\infty \right)=o\left(r^{*}\right)\geq o\left(r\right),$$

which proves (ii).□

The second part of Proposition 2.2 implies the existence of a finite interval $\left[0,r^{*}\right]$ of relevant set parameters; increasing the set parameter beyond $r^{*}$ leaves the optimal designs as well as the optimal value of the δ‐criterion unchanged. We will call any such $r^{*}$ a *set upper bound*.

Algorithm 1 provides a simple iterative method of computing $r^{*}$. Our experience shows that it usually requires only a small number of recomputations of the $\delta_{r}$‐optimal design, even if $r_{ini}$ is small and *q* is close to 1, resulting in a good set upper bound $r^{*}$ (see the metacode of Algorithm 1 for details). Due to the high speed and stability of the computation of the values of $\delta_{r}$ for candidate designs, it is possible to use an adaptation of the standard KL exchange heuristic to compute the input value o(∞), as well as to obtain $\delta_{r}$‐optimal designs in steps 2 and 9 of the algorithm itself.

Algorithm 1 **A simple algorithm for computing a set upper bound**

**Table:** 

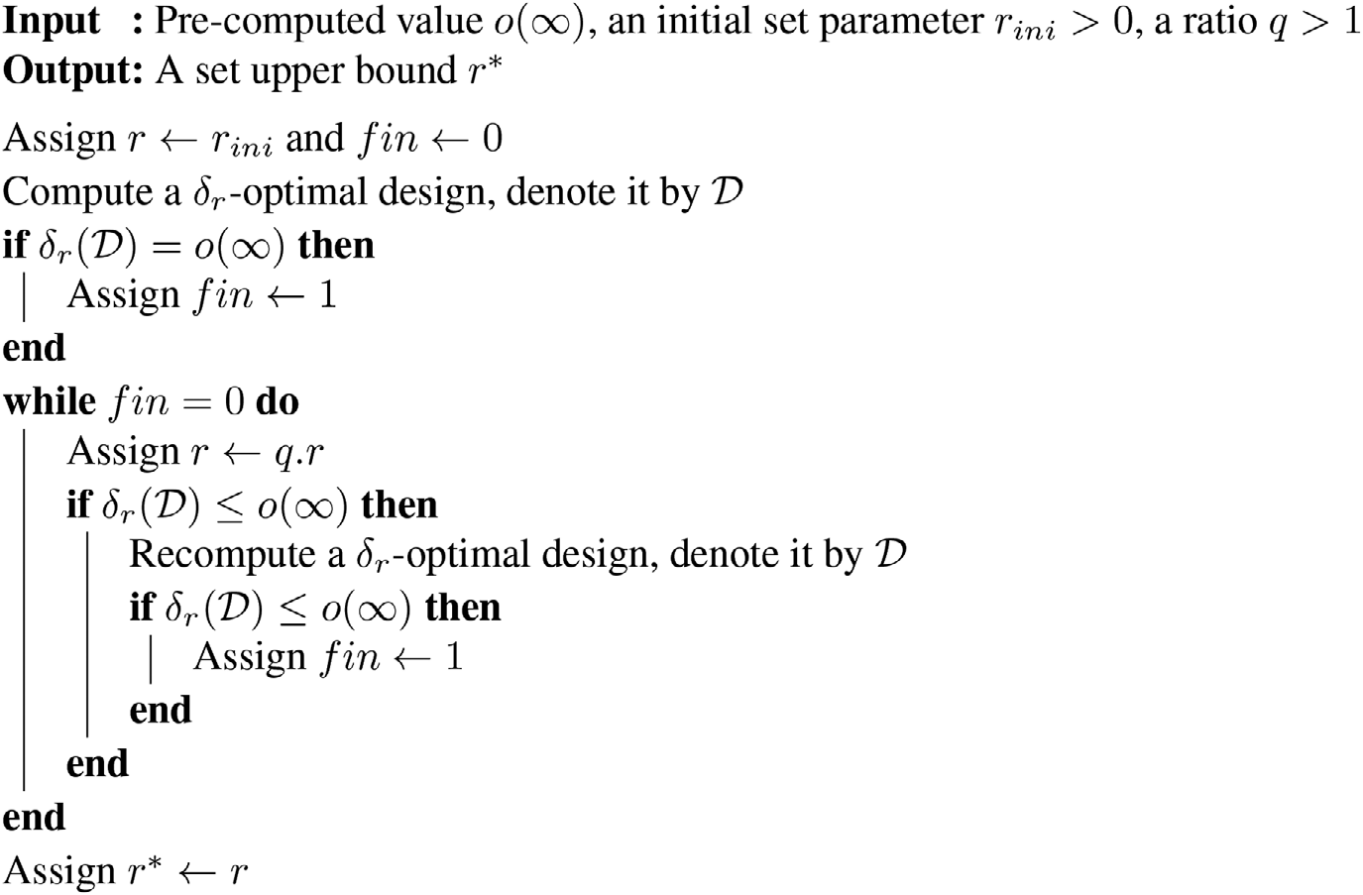

### 2.4 Example 1 continued

**Figure 3:**
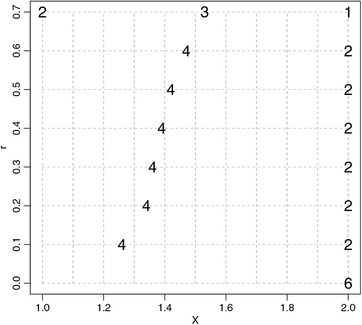
$\delta_{r}$‐Optimal designs of size n=6 for different *r*s; see the second part of the motivating example. The horizontal axis corresponds to the design space, and the vertical axis corresponds to different spans *r* of the flexible nominal sets. For each *r*, the figure displays the number of repeated observations at different design points, corresponding to the $\delta_{r}$‐optimal design

Consider the models from the motivating example. Let X={1.00,1.01,…,2.00}, ${\tilde{\theta }}_{0}=e$, and ${\tilde{\theta }}_{1}=1$. Note that these nominal values satisfy $\eta_{0}\left({\tilde{\theta }}_{0},1\right)=\eta_{1}\left({\tilde{\theta }}_{1},1\right)$. Moreover, let us set ${\tilde{\Theta }}_{0}^{\left(1\right)}=\left[e-1,e+1\right]$ and ${\tilde{\Theta }}_{1}^{\left(1\right)}=\left[0,2\right]$, and let the required size of the experiment be n=6. First, we computed the value $o^{2}\left(\infty \right)\approx 0.02614$. Next, we used Algorithm 1 with $r_{ini}=0.3$ and $q=1+10^{-6}$, which returned a set upper bound $r^{*}\approx 0.6787$ after as few as seven computations of $\delta_{r}$‐optimal designs. Informed by $r^{*}$, we computed $\delta_{r}$‐optimal designs for r=0.01,0.1,0.2,…,0.7. The resulting $\delta_{r}$‐optimal designs are displayed in Figure 3. Note that if ${\tilde{\Theta }}^{\left(r\right)}$s are very narrow, the $\delta_{r}$‐optimal design is concentrated in the design point x=2, effectively maximizing the difference between $\eta_{0}\left({\tilde{\theta }}_{0},x\right)$ and $\eta_{1}\left({\tilde{\theta }}_{1},x\right)$. For larger values of *r*, the $\delta_{r}$‐optimal design has a 2‐point and ultimately a 3‐point support.

For some pairs of competing models there exists a set upper bound $r^{*}$, beyond which the values of $\delta_{r}$ are constantly 0 for all designs. These cases can be identified by solving a linear programming (LP) problem, as we show next.

Proposition 2.3 Let $\bar{D}$ be the design that performs exactly one trial in each point of X. Consider the following LP problem with variables r∈R, $\theta_{0}\in R^{m}$, $\theta_{1}\in R^{m}$:

(14) $$\begin{array}{ccc} \min & & r\\ s.t. & & F_{0}\left(\bar{D}\right)\theta_{0}+a_{0}\left(\bar{D}\right)=F_{1}\left(\bar{D}\right)\theta_{1}+a_{1}\left(\bar{D}\right),\\ & & \theta_{0}\in {\tilde{\Theta }}_{0}^{\left(r\right)},\;\theta_{1}\in {\tilde{\Theta }}_{1}^{\left(r\right)},\;r\geq 0. \end{array}$$

Assume that (14) has some solution, and denote one solution of (14) by ${\left(r^{*},\theta_{a}^{T},\theta_{b}^{T}\right)}^{T}$. Then, $r^{*}$ is a finite set upper bound. Moreover, o(r)=0 for all $r\in [r^{*},\infty ]$.

From the expression (7) we see that for any design D and its nonreplication version $D^{nr}$ we have $\delta_{r}\left(D^{nr}\right)=0$ implies $\delta_{r}\left(D\right)=0$. Moreover, if $D_{2}⪰D_{1}$ in the sense that $D_{2}$ is an augmentation of $D_{1}$ then $\delta_{r}\left(D_{2}\right)=0$ implies $\delta_{r}\left(D_{1}\right)=0$. Now let ${\left(r^{*},\theta_{a}^{T},\theta_{b}^{T}\right)}^{T}$ be a solution of (14), let $r\geq r^{*}$ and let D be any design. Definition of $\delta_{r}$ and the form of (14) imply $\delta_{r}\left(\bar{D}\right)=0$. From $\bar{D}⪰D^{nr}$ we see that then $\delta_{r}\left(D^{nr}\right)=0$, hence $\delta_{r}\left(D\right)=0$. The proposition follows.□

Note that $r^{*}$ obtained using Proposition 2.3 does not depend on *n*, that is, it is a set upper bound simultaneously valid for all design sizes. The basic discriminability condition implies that $r^{*}\neq 0$.

If the competing models are linear, vectors $a_{0}\left(\bar{D}\right)$ and $a_{1}\left(\bar{D}\right)$ are zero. Therefore, (2.3) has a feasible solution ${\left(r,0_{m}^{T},0_{m}^{T}\right)}^{T}$ for any r≥0 such that both ${\tilde{\Theta }}_{0}^{\left(r\right)}$ and ${\tilde{\Theta }}_{1}^{\left(r\right)}$ cover $0_{m}$. That is, for the case of linear models, there is a finite set upper bound $r^{*}$ beyond which the $\delta_{r}$‐values of all designs vanish. However, the same holds for specific nonlinear models, including the ones from Section 3.

Proposition 2.4 Assume that both competing regression models are linear provided that we consider a proper subset of their parameters as known constants. Then (2.3) has a finite feasible solution, that is, there exists a finite set upper bound $r^{*}$ such that o(r)=0 for all $r\in [r^{*},\infty ]$.

Without loss of generality, assume that fixing the first $k_{0}<m$ components of θ_0_ converts Model 0 to a linear model. More precisely, let $\theta_{01},\ldots ,\theta_{0m}$ denote the components of θ_0_ and assume that

$$\eta_{0}\left(\theta_{0},x\right)=\sum_{j=k_{0}+1}^{m}\gamma_{j}^{\left(0\right)}\left(\theta_{01},\ldots ,\theta_{0k_{0}},x\right)\theta_{0j}$$

for some functions $\gamma_{j}^{\left(0\right)}$, $j=k_{0}+1,\ldots ,m$. Choose ${\hat{\theta }}_{0}$ such that ${\hat{\theta }}_{0j}={\tilde{\theta }}_{0j}$ for $j=1,\ldots ,k_{0}$, and ${\hat{\theta }}_{0j}=0$ for $j=k_{0}+1,\ldots ,m$. Make an analogous assumption for Model 1 and also define ${\hat{\theta }}_{1}$ analogously. It is then straightforward to verify that for the design $\bar{D}$ from Proposition 2.3 we have $F_{k}\left(\bar{D}\right){\hat{\theta }}_{k}+a_{k}\left(\bar{D}\right)=0_{d}$, where d=#X, for both k=0,1. Therefore, any ${\left(r,{\hat{\theta }}_{0}^{T},{\hat{\theta }}_{1}^{T}\right)}^{T}$ such that ${\hat{\theta }}_{0}\in {\tilde{\Theta }}_{0}^{\left(r\right)}$ and ${\hat{\theta }}_{1}\in {\tilde{\Theta }}_{1}^{\left(r\right)}$ is a solution of (14) in Proposition 2.3.□

In the following, we numerically demonstrate that the δ design criterion leads to designs which yield a high probability of correct discrimination.

## 3 AN APPLICATION IN ENZYME KINETICS

This real applied example is taken from Bogacka, Patan, Johnson, Youdim, and Atkinson (2011) and was already used in Atkinson (2012) to illustrate model‐discrimination designs. There two types of enzyme kinetic reactions are considered, where the reactions velocity *y* is alternatively modeled as

(15) $$y=\frac{\theta_{01}x_{1}}{\theta_{02}\left(1+\frac{x_{2}}{\theta_{03}}\right)+x_{1}}+\varepsilon$$

and

(16) $$y=\frac{\theta_{11}x_{1}}{\left(\theta_{12}+x_{1}\right)\left(1+\frac{x_{2}}{\theta_{13}}\right)}+\varepsilon ,$$

which represent competitive and noncompetitive inhibition, respectively. Here *x*_1_ denotes the concentration of the substrate and *x*_2_ the concentration of an inhibitor. The data used in Bogacka et al. (2011) from an initial experiment of 120 observations are on Dextrometorphan–Sertraline and yields the estimates displayed in Table 1, where Gaussian errors were assumed. Assumed parameter spaces were not explicitly given there, but can be inferred from their figures as $\theta_{0,1},\theta_{1,1}\in \left(0,\infty \right)$, $\theta_{0,2},\theta_{1,2}\in \left(0,60\right]$, and $\theta_{0,3},\theta_{1,3}\in \left(0,30\right]$, respectively. Designs for parameter estimation in these models were recently given in Schorning, Dette, Kettelhake, and Möller (2017).

**Table 1:** Parameter estimates and corresponding standard errors for models (15) and (16), respectively

	Estimate $\hat{\theta }$	*SE* ${\hat{\sigma }}_{\theta }$			Estimate $\hat{\theta }$	*SE* ${\hat{\sigma }}_{\theta }$
θ_01_	7.298	0.114		θ_11_	8.696	0.222
θ_02_	4.386	0.233		θ_12_	8.066	0.488
θ_03_	2.582	0.145		θ_13_	12.057	0.671

In Atkinson (2012), the two models are combined into an encompassing model:

(17) $$y=\frac{\theta_{21}x_{1}}{\theta_{22}\left(1+\frac{x_{2}}{\theta_{23}}\right)+x_{1}\left(1+\frac{\left(1-\lambda \right)x_{2}}{\theta_{23}}\right)}+\varepsilon ,$$

where λ=1 corresponds to (15) and λ=0 to (16), respectively. Following the ideas of Atkinson (1972) as used, for example, in Atkinson (2008) or Perrone, Rappold, and Müller (2017) one can then proceed to find so‐called $D_{s}$‐optimal (i.e., D‐optimal for only a subset of parameters) designs for λ and employ them for model discrimination. Note that also this method is not fully symmetric as it requires a nominal value for λ for linearization of (17), which induces some kind of weighting.

The nominal values used in Atkinson (2012) obviously motivated by the estimates of (15) were ${\tilde{\theta }}_{01}={\tilde{\theta }}_{11}={\tilde{\theta }}_{21}=10$, ${\tilde{\theta }}_{02}={\tilde{\theta }}_{12}={\tilde{\theta }}_{22}=4.36$, ${\tilde{\theta }}_{03}=2.58$, ${\tilde{\theta }}_{13}=5.16$, and ${\tilde{\theta }}_{23}=3.096$. However, note that particularly for model (16) the estimates in Table 1 give considerably different values and also nonlinear least squares directly on (17) yields the deviating estimates given in Table 2. The design region used was rectangular $X=X_{1}\times X_{2}=\left[0,30\right]\times \left[0,40\right]$.

**Table 2:** Parameter estimates and corresponding standard errors for the encompassing model (17)

	Estimate $\hat{\theta }$	*SE* ${\hat{\sigma }}_{\theta }$
θ_21_	7.425	0.130
θ_22_	4.681	0.272
θ_23_	3.058	0.281
λ	0.964	0.019

In Table 2 of Atkinson (2012) four approximate optimal designs (we will denote them as A1–A4) were presented: the T−optimal designs assuming λ=0 (A1) and λ=1 (A4), a compound *T*‐optimal design (A3), and a $D_{s}$‐optimum (A2) for the encompassing model (for the latter note that Atkinson assumed λ=0.8, whereas the estimate suggest a much higher value). We will compare our δ‐optimal designs against exact versions of these designs, properly rounded by the method of Pukelsheim and Rieder (1992).

### 3.1 Confirmatory experiment n=6, normal errors

Let us first assume we want to complement the knowledge from our initial experiment by another experiment for which, however, we were given only limited resources, for example, for the sample sizes of only n=6 observations. Note that the aim is not to augment the previous 120 observations but to make a confirmatory decision just using the new observations. That is we are using the data from the initial experiment just to provide us with nominal values for parameter estimates and noise variances for the simulation, respectively. This is a realistic scenario if for instance for legal reasons the original data had to be deleted and only summary information was available.

As we are assuming equal variances for the two models we are using the estimate for the error standard deviation $\hat{\sigma }=0.1526$ from the encompassing model as a base value for the simulation error standard deviation. However, using $\hat{\sigma }$ was not very revealing for the discriminatory performance was consistently high for all designs. Thus, to accentuate the differences the actual standard deviation used was $2\times \hat{\sigma }$ instead (unfortunately an even higher inflation is not feasible as it would result in frequent negative observations leading to faulty ML‐estimates). We then simulated the data‐generating process under each model for N=10000 times and calculated the total percentages of correct discrimination (hit rates) when using the likelihood ratio as decision rule.

**Figure 4:**
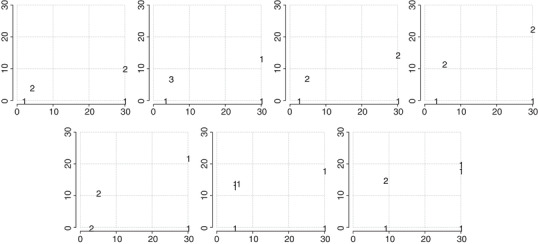
Compared designs: first row A1–A4, second row δ1–δ3

We are comparing the designs A1–A4 to three specific δ designs δ1,δ2, and δ3, which represent a range of different nominal intervals. Specifically we chose ${\tilde{\Theta }}_{k}=\left[{\tilde{\theta }}_{k1}\pm r{\tilde{\sigma }}_{k1}\right]\times \left[{\tilde{\theta }}_{k2}\pm r{\tilde{\sigma }}_{k2}\right]\times {\left[{\tilde{\theta }}_{k3}\pm r{\tilde{\sigma }}_{k3}\right]}_{k=0,1}$, where we chose ${\tilde{\theta }}_{kj}={\hat{\theta }}_{kj}$ and ${\tilde{\sigma }}_{kj}={\hat{\sigma }}_{kj}$ for k=0,1 and j=1,2,3. The tuning parameter *r* was set to three levels: r=1 (which is close to the lower bound of still providing a regular design), r=5 and r=15 (which is sufficiently close to the theoretical upper bound to yield a stable design), respectively. To make the latter more precise: the models in considerations are such that if we fix the last two out of the three parameters, then they become one‐parameter linear models. Therefore, using Proposition 2.4 we know that there exists a finite set upper bound $r^{*}$. Solving (14) provides the numerical value $r^{*}\approx 64.02$. Note that the same bound is valid for all design sizes *n*. Designs A1–A4 and δ1 all contain four support points, while δ2 has six and δ3 has five, respectively. A graphical depiction of the designs is given in Figure 4.

*Robustness study*: As we would like to avoid comparing designs only when the data are generated from the nominal values (although this favors all designs equally), we perturbed the data‐generating process by drawing parameters from uniform distributions drawn at $\tilde{\theta }\pm c\times {\tilde{\sigma }}_{\theta }$, where *c* then acts as a perturbation parameter. Under these settings all these designs fare pretty well as can be seen from Table 3. However, A4 and δ2 seem to outperform the other competing designs by usually narrow margins except perhaps for A1, which is consistently doing worst. Note that in a real situation the true competitors of δ‐optimal designs are just A2 and A3 as it is unknown beforehand which model is true.

**Table 3:** Total hit rates for N=10000 under each model, maximal values in boldface

c	0		1		5	
True model	η_0_	η_1_	η_0_	η_1_	η_0_	η_1_
A1	91.11	94.45	91.35	93.95	90.44	93.24
A2	97.11	96.75	97.47	96.64	96.74	96.27
A3	96.60	96.51	96.47	96.40	95.69	96.06
A4	**97.94**	96.57	97.73	96.29	97.62	96.07
δ1	97.59	95.11	97.43	94.90	**97.71**	94.56
δ2	97.93	**97.03**	**97.77**	**96.67**	97.20	**96.54**
δ3	96.50	95.29	96.42	95.36	96.19	95.64

### 3.2 A second large‐scale experiment n=60, log‐normal errors

As the discriminatory power of all the designs for n=60 is nearly perfect, we are required to inflate the error variance. However, using additive normal errors in the data‐generating process and inflating the variance by a large enough factor, would generate a large number of negative observations, which renders likelihood estimation invalid. So, the data‐generating process was adapted to use multiplicative log‐normal errors. The observations were then rescaled to match the means from the original process. This way we are at liberty to inflate the error variance by any factor without producing faulty observations. Note that now the data‐generating process does not fully match the assumptions under which the designs were generated, but this can just be considered an extended robustness study as it holds for all compared designs equally. We could of course also have calculated the designs under the same data‐generating process, but as the fit of the model to the original data is not greatly improved and models (15) and (16) seem firmly established in the pharmacological literature, we refrained from doing this.

Perturbation of the parameters here did not exhibit a discernible effect, while the error inflation still does. For brevity, we here report only again the results for using $5\times \hat{\sigma }$ (and c=0). The respective designs δ1–3 were qualitatively similar to those given in Figure 4 albeit with more diverse weights. In this simulation we generated 100 instances of n=60 observations from these designs a thousand times.

**Figure 5:**
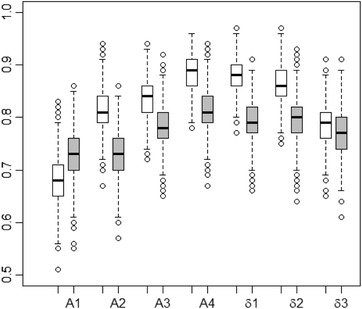
Boxplot for the total correct classification rates for all designs using nominal values and error standard deviations of $5\times \hat{\sigma }$; white under η_0_, grey under η_1_

The corresponding boxplots of the correct classification rates are given in Figure 5. In this setting A4 seems a bit superior even under η_1_ (remember it being the *T*‐optimum design assuming η_0_ true), while δ1 and δ2 come close (and beat the true competitors A2 and A3) with A1 again being clearly the worst.

## 4 CONCLUSIONS AND DIRECTIONS FOR FURTHER RESEARCH

We have presented a novel design criterion for symmetric model discrimination. Its main advantage is that design computations, unlike for *T*‐optimality, can be undertaken with efficient routines of quadratic optimization that in general enhance the speed of computations by an order of magnitude. An optimal exact design problem is a problem of discrete optimization, and the efficiency of its solution critically depends on the speed of evaluation of the design criterion. By a series of approximations, we substituted the theoretically ideal but numerically infeasible computation of the probability of correct discrimination with a simple convex optimization, which can be solved rapidly and reliably. Combined with the proposed methodology of flexible nominal sets, we can construct an entire sequence of exact experimental designs efficient for discrimination between models. Also it was shown in an example that resulting designs are competitive in their actual discriminatory abilities.

The notion of flexible nominal sets may have independent merit. Note again the distinction between parametric spaces and flexible nominal sets (and thus the principal distinction to “rigid” minimax approaches). Parametric spaces usually encompass all theoretically possible values of the parameters, while flexible nominal sets can contain the unknown parameters with very high likelihood, and still be significantly smaller than the original parameter spaces. In this paper, we do not completely specify the process of constructing the flexible nominal sets, but if we perform a two stage experiment, with a second, discriminatory phase, the potential specification through confidence intervals is an important problem.

As the approach suggested offers a fundamentally new way of constructing discriminatory designs, many properties are yet unexplored. A nonexhaustive list of questions follows.

*Sequential procedure*. The proposed method lends itself naturally to a two‐stage procedure, where parameter estimates and confidence intervals are employed as nominal values in the second stage. Even sequential generation of design points can be straightforwardly implemented.

*Approximate designs*. Proposition 2.1 is a possible gateway for the development of the standard approximate design theory for δ‐optimality because the criterion $\delta_{app}^{2}$ is concave on the set of all approximate designs. Therefore, it is possible to work out a minimax‐type equivalence theorem for δ‐optimal approximate designs, and use specific convex optimization methods to find a δ‐optimal approximate designs numerically. For instance, it would be possible to employ methods analogous to Burclová and Pázman (2016) or Yue, Vandenberghe, and Wong (2018).

*Utilization of the* δ*‐optimal designs for related criteria*. For a design $D=(x_{1},\ldots ,x_{n})$, a natural criterion closely related to $\delta_{r}$‐optimality can be defined as

$$\begin{array}{c} {\tilde{\delta }}_{r}\left(D\right)=\underset{\theta_{0}\in {\tilde{\Theta }}_{0}^{\left(r\right)},\theta_{1}\in {\tilde{\Theta }}_{1}^{\left(r\right)}}{\inf}\tilde{\delta }\left(D|\theta_{0},\theta_{1}\right),\;\text{where}\;\\ \tilde{\delta }\left(D|\theta_{0},\theta_{1}\right)=\left\|{\left(\eta_{0}\left(\theta_{0},x_{i}\right)\right)}_{i=1}^{n}-{\left(\eta_{1}\left(\theta_{1},x_{i}\right)\right)}_{i=1}^{n}\right\|. \end{array}$$

The criterion ${\tilde{\delta }}_{r}$ requires a multivariate nonconvex optimization for the evaluation in each design D, which entails possible numerical difficulties and a long time to compute an optimal design. However, the $\delta_{r}$‐optimal design, which can be computed rapidly and reliably, can serve as efficient initial design for the optimization of ${\tilde{\delta }}_{r}$. Note that if ${\tilde{\Theta }}_{0}$ is a singleton containing only the nominal parameter value for Model 0, the $\delta_{r}$‐optimal designs could potentially be used as efficient initial designs for computing the exact version of the criterion of *T*‐optimality.

*Selection of the best design from a finite set of possible candidates*. As most proposals for the construction of optimal experimental designs, the method depends on the choice of some tuning parameters or even on entire prior distributions (in the Bayesian approach), which always results in a set of possible designs. It would be interesting to develop a comprehensive Monte‐Carlo methodology for the choice of the best design out of this pre‐selected small set of candidate designs. A useful generalization of the rule would take into account possibly unequal losses for the wrong classification.

*Noncuboid sets*. The methodology could certainly be extended to other types of flexible nominal sets, particularly when we are interested in functional relations among the parameters. However, then the particularly efficient box constrained quadratic programming algorithm could not be utilized.

*Higher order approximations*. As a referee remarked it is possible to employ tighter approximations of the sets of mean values of responses than the one that we suggest. For instance, it would be possible to use the local curvature of the mean‐value function. However, this may also lead to the loss of numerical efficiency of the method.

*More than two rival models*. Another referee remark leads us to point out the natural extension to investigate a weighted sum or the minimum δ over all paired comparisons. The implications of these suggestions, however, requires deeper investigations.

*Different error variances*. Yet another referee requested a clarification for how to proceed in case of unequal error variances for the two models. In case the functional form of these variances are known simple standardizations of the models will suffice. All other cases, including dependencies of the errors, will require more elaborate strategies.

*Combination with other criteria*. The proposed method can produce poor or even singular designs for estimating model parameters. Because of this problem, already mentioned in Atkinson and Fedorov (1975), Atkinson (2008) used a compound criterion called DT‐optimality. The same approach is possible for δ‐optimality. However, our numerical experience suggests that for a large enough size of the flexible nominal set, the δ‐optimal designs tend to be supported on a set that is large enough for estimability of the parameters, without any combination with an auxiliary criterion. A detailed analysis goes beyond the scope of this paper.

## CONFLICT OF INTEREST

The authors declare that there is no conflict of interest.

### Open Research Badges

This article has earned an Open Data badge for making publicly available the digitally‐shareable data necessary to reproduce the reported results. The data is available in the Supporting Information section.

This article has earned an open data badge “**Reproducible Research**” for making publicly available the code necessary to reproduce the reported results. The results reported in this article could fully be reproduced.

## Supporting information

SUPPORTING INFORMATION
